# Thank you to *The Lancet Regional Health – Europe*'s clinical and statistical peer reviewers in 2025

**DOI:** 10.1016/j.lanepe.2026.101608

**Published:** 2026-01-30

**Authors:** Pooja Jha, Ivana Nedic, Stephanie Becker, Anna Johnson

**Affiliations:** The Lancet Regional Health – Europe, Munich, Germany

Peer review is the cornerstone of scientific publishing, relying on the expertise, judgement, and generosity of researchers who contribute their time to the evaluation of others’ work. As we reflect on peer review at *The Lancet Regional Health – Europe* in 2025, we extend our sincere thanks to the clinical and statistical reviewers whose contributions have been integral to the quality and integrity of the journal.

In 2025, 1305 reviewers reviewed for the journal. This represents continued growth from 1192 reviewers in 2024 and 917 reviewers in 2023, an increase of more than 40% over 2 years. This increase reflects both the rising volume and breadth of submissions received by the journal and the willingness of the research community to engage in the demanding and often invisible work of peer review. We acknowledge that this contribution comes alongside reviewers’ own research, clinical, and teaching responsibilities, and we are deeply grateful for their commitment.

Gender diversity among reviewers remains an important area of focus. In 2025, 57.6% of reviewers identified as men (up from 56.7% in 2024), 32.9% identified as women (down from 34.6%), 0.7% identified as non-binary or gender diverse (largely unchanged from 0.8%), and 8.8% chose not to disclose their gender identity (up from 8.0%). Taken together, these data indicate no improvement in gender balance in 2025, underscoring the need for renewed and more targeted efforts to address persistent structural barriers within academic peer review.

Geographical representation continues to reflect broader global inequities in research participation. Although the majority of our reviewers (92.4%) were from high-income countries, this proportion has decreased from 93.2% in 2024. We are encouraged to see contributions from low-income and middle-income countries (LMICs) increased from 5.5% in 2024 to 6.0% in 2025, although participation from LMICs remains limited.

Our reviewers were from 62 countries, compared with 55 countries in 2024, reflecting our expanding reach. The largest numbers of reviewers were based in the UK, the USA, Italy, Germany, Australia, France, and the Netherlands. We will continue to intensify efforts to broaden reviewer representation from under-represented regions in the years ahead.

Within Europe, regional imbalances in reviewer representation persist. In 2025, reviewers based in Eastern Europe accounted for approximately 1–2% of the total reviewer pool and around 3% of reviewers based in Europe. These low proportions likely reflect, at least in part, the comparatively small number of submissions received from eastern European countries. Nonetheless, as a pan-European journal, we recognise the importance of strengthening engagement with researchers across all parts of the region to ensure that diverse health system contexts and perspectives are adequately represented within the peer review process.

As part of ongoing efforts to improve transparency and inclusivity in peer review, optional Equity, Diversity, and Inclusion (EDI) personal classifications have been available to reviewers across *The Lancet* journals since September, 2024 (see margin description for EDI classification). Users can voluntarily assign these EDI classifications to submitted manuscripts or to their individual accounts to indicate their expertise. These classifications span a wide range of areas, including health equity and discrimination in health. In 2025, 2.7% of reviewers across *The Lancet* group who completed at least one review selected at least one EDI classification, and within *The Lancet Regional Health – Europe*, 1.8% of reviewers selected at least one EDI classification. Considering that many submissions and reviews are not directly related to these topics, these figures should be understood as a baseline measure of engagement. As these classifications continue to be adopted by reviewers, they provide an important reference point from which to monitor engagement and to inform future efforts to strengthen inclusive participation in peer review.

Across the past 3 years, these data illustrate that while expanding the reviewer pool is achievable, meaningful improvements in diversity are more gradual and challenging. Growth alone is insufficient; it must be accompanied by deliberate and sustained strategies to broaden participation across gender and geography. As editors, we remain committed to identifying, inviting, and supporting reviewers from under-represented groups and regions, while maintaining the high standards expected of peer review for *The Lancet* group.

Peer reviewers play a fundamental role in safeguarding the credibility of scientific publishing. Once again, we extend our profound thanks to all reviewers who supported *The Lancet Regional Health – Europe* in 2025. Your contributions remain essential to the journal's mission, and we look forward to continuing this work together in the years ahead.

Elahe Abbaspour

Tom Abbott

Hilal Abdessamad

Renata Abrahao

Lucas Guimarães Abreu

Karim Abu-Omar

Lisa Adams

Tracie Afifi

Mark Agulnik

Charles Agyemang

Amy Louise Ahern

Raheel Ahmed

Agneta Akesson

Dilara Akhoundova

Ibrahim Akin

Vance Albaugh

Peter Albertsen

Markus Albertsmeier

Werner Albrich

Krystallenia Alexandraki

Paul R Algra

Ana Margarida Alho

Shehzad Ali

Gianfranco Alicandro

Moran Amit

Antonio J Amor

Atul Anand

Mads Andersen

Spinello Antinori

Gema Ariceta

Albert Ariza Sole

Elizabeth V Arkema

Wilbert S Aronow

Yasuhide Asaumi

David Asboe

Ryo Ashida

Shiu Lun Au Yeung

Heinrich Audebert

Segolene Ayme

Xavier Ayrignac

Taj Azarian

Luciano Azevedo

Élie Azria

Ganesh M Babulal

Helena Backman

Elizabeth Badley

Houda Bahig

Shasha Bai

R Peter Bailey

Sébastien Bailly

Stephen Bain

Olusegun Baiyewu

Farhad Bakhtiary

Maciej Banach

Eugenio Baraldi

Angelo Barbato

Sally Barber

Louise Barbier

Edit Bardi

Marc Bardou

Giuseppe Barisano

Frederik Barkhof

Ruanne Barnabas

Alexandra Barratt

Jenny Barrett

Alessandro Bartoloni

Alfred Bartolucci

Rupert Bartsch

Melanie A Barwick

Buddha Basnyat

Quique Bassat

Sarah Bates

Nicole Baumann

Peter Becher

Paul Beggs

Rinad S Beidas

Katy Bell

David Benton

Roberto Bergamaschi

Mette M Berger

Johannes Berkhof

Paola Bertuccio

Friedhelm Beyersdorf

Julien Bezin

Vineet Bhandari

Aneel Bhangu

Vishal Bhavsar

Anja Bieber

Dan Bieler

Merijn Bijlsma

Laurent Billot

Paolo Bironzo

Johan Bjureberg

Isobel Blake

Julie Blanc

Flora Blangis

Juan Antonio Blasco-Amaro

Jean-Yves Blay

Katherine Bline

Bertil Blok

Christopher Blyth

Irina Diana Bobolea

Stefania Boccia

Petri Böckerman

Aurelie Bocquier

Jacob Bodilsen

Yuliya Bodryzlova

Jolanda M A Boer

Marja A Boermeester

Christoph Boesecke

Giorgio Bogani

Marialaura Bonaccio

Michela Bonafede

Francesco Bonella

Udo Bonnet

Daniela Bonofiglio

Cécile R L Boot

Mark Bosmans

Paulo Alexandre Boto

Thierry Boulain

Arnaud Bourdin

Jacqueline Bowden

Peter Bower

Oksana R Boyarchuk

Chiara Braconi

Caroline Bradbury-Jones

Carolyn Bramante

Axel Brandes

Christian T Brandt

Ola Bratt

Alexandra Bražinová

Susanne Breitner

Viviane Bremer

Margaret M Brennan

Patricia Brennan

Keno Bressem

Liz Brewster

Amy Brodtmann

Jeffrey Reynold Brubacher

Ronny Bruffaerts

Adam Brufsky

Christian J L Brun-Buisson

Raffaele Brustia

Marie Bryce

Gabriel Bsteh

Christoph Buck

Nicholas Buckley

Stefan Buettner

Raffaele Bugiardini

Nóra Bunford

Claudia Buntrock

Danilo Buonsenso

Fiona Burns

Patrizia Burra

Luca Busani

Reinhard Busse

Gary Butler

Joaquin Cabezas

Guoen Cai

Xiaoling Cai

Yutong Samuel Cai

Philip Calder

Simona Calugi

Xavier Calvet Calvo

Cristina Calvo

Gonzalo Calvo

Sharon Cameron

Calogero Camma

Jackie Campbell

Michael Campbell

Marjo Campmans-Kuijpers

Corrado Campochiaro

Silva Canetto

Antonio Cannatà

Bin Cao

Davide Cao

Riccårdo Capocaccia

Flavio Caprioli

David Carabantes Alarcón

Helena Carreira

Laura Carroll

Claire Carson

Alice S Carter

Ben Carter

Jordi Casabona

Maria Casanova

Rosa Casas

Antonio Cascio

Francesco Castelli

Rafael Castro Delgado

Paola Cavalla

Francesca Cecchi

Luca Cegolon

Leo Anthony Celi

Edina Cenko

Jad Chahoud

Martin Chalumeau

Wing Chung Chang

Emmanuel Chartier-Kastler

Mary Lou Chatterton

Rekha Chaudhuri

Minglong Chen

Mu-Hong Chen

Qingui Chen

Sitong Chen

Xiqun Chen

Yingyao Chen

Yunbo Chen

Jeanie Cheong

Ching-Lung Cheung

Li Cheung

Eusebi Chiner

Adriano Chiò

Ovidiu Chioncel

Kelvin Choi

Bernard Cholley

Shu-Ling Chong

Suma Choorapoikayil

Jakob Christensen

Oriana Ciani

Massimo Ciccozzi

Arrigo F G Cicero

Simon Cichosz

Irfan Cicin

Catia Cilloniz

Olivier Claris

David Clark

Sean Clouston

Andrew J S Coats

Ohad Cohen

Lauren Collins

Simon Collins

Philippe Colson

Pierfranco Conte

Brian Conway

Andrew Copas

Massimiliano Copetti

Louise Corben

Alberto Cordero

Oliver Cornely

Rosie Cornish

Giovanni Corso

Samuele Cortese

Paolo Angelo Cortesi

Tina Costacou

Paul Cottu

Marcello Covino

Patricia Coyle

Deborah L Cragun

Paul Crawshaw

Bruno Crestani

Michael Crooks

Natividad Cuende

Marco Cusini

Maarten Cuypers

Darlan Da Silva Candido

Omid Dadras

Andrei Dadu

Michael Dalager-Pedersen

Philipp Dammann

Goodarz Danaei

Antonio Daniele

Anna-Karin Danielsson

M Carolina Danovaro-Holliday

Pojsakorn Danpanichkul

Lynne Dawkins

Fatimah Dawood

Andrew Day

Geertruida de Bock

Giovanni de Gaetano

Sabine de Greeff

Xavier de Lamballerie

Carlos De las Cuevas

Ruggero De Maria

Charles de Mestral

Etienne de Montmollin

Anniek de Ruijter

Tom Decroo

Suzette Delaloge

Philippe Delespaul

Julio Delgado

Victoria Delgado

Sarah Dellière

Clément Delmas

Nele Demeyere

Salil Deo

Birgit Derntl

Olivier M Detry

Brecht Devleesschauwer

Roland Devlieger

Ketan Dhatariya

Stefania Angela Di Fusco

Francesco Di Gennaro

Massimo Di Maio

Antonio Di Meglio

Esperanza Diaz Perez

Paul Dickman

Joseph Dieleman

Jutta Dierkes

Anelia Dietmann

Pablo Díez-Villanueva

Dobromir Dimitrov

Guglielmo Dini

Diana Maria DiNitto

Eveline Dirinck

Antonino Ditto

Matthew Dodd

Richard Dodel

Hiten Dodhia

Torsten Doenst

Ing-Mari Dohrn

Christiane Dolecek

Geert Dom

Javier Domínguez-Ortega

Alexander Domnich

Elise G P Dopper

Alex Dregan

Joanne M Droney

Amanda Drury

Sarah M Dry

Xianglin Du

Brett Duane

Ann-Sofi Duberg

Andrew Duckworth

Amylou Dueck

Carole Dufouil

Daphne Dumoulin

Ralf Dürrwald

Lauren Mc Carl Dutra

Angel Dzhambov

Kristie Ebi

Adrian Edwards

Terje Eikemo

Nada El Husseini

Hadi Emamat

Hedley Emsley

Ingunn Marie Stadskleiv Engebretsen

Gerda Engholm

Charli Eriksson

Sylvie Escolano

Isaac Estevan

Catherine Ettman

Patricia Eustachio Colombo

Gian Paolo Fadini

Martin Faehling

Björn H Falkenburger

Amary Fall

Weijia Fang

Eric Faragher

Dimitrios Farmakiotis

Forough Farrokhyar

Seena Fazel

Jerome Jeffrey Federspiel

Joerg Fegert

Zhiqiang Feng

Lyn D Ferguson

Neil Ferguson

Romulo Fernandes

Adolfo Fernandez

Óscar Fernández

Núria Fernández-Hidalgo

Clarissa Ferrari

Gerasimos Filippatos

Massimo Filippi

Elizabeth Finger

Fabian Finkelmeier

Paolo Fiorina

Stefan Flasche

Kelly Flemming

Andrew Fogarty

Slim Fourati

Anne Marie France

Francesco Franceschi

Kate Frazer

Lucinda Freeman

Joseph Friedman

Douglas R Galasko

Thomas C E Gale

Michel Galinier

Jennifer Gallagher

James Galloway

Silvano Gallus

Shi-Rui Gan

Marialuisa Gandolfi

Ron Gansevoort

Elena García-Martín

Anthony Garcia-Prats

Bradley Gardiner

Nicolas Garin

Jérôme Garot

Kirk Garratt

Jerzy Gąsowski

Anna Gavin

Houssein Gbaguidi-Haoré

Zongyuan Ge

Vittorio Gebbia

Marco Geraci

Jade Ghosn

Elisa Giallongo

Diana Giannarelli

Eric Giannoni

Katherine Gibney

Johannes Giesinger

Miguel Gil Gil

Yvonne Gilleece

Paolo Giorgi Rossi

Marta Giovanetti

Gavin Giovannoni

Gunnar Gislason

Mika Gissler

Heide Glaesmer

Stanton Glantz

Deniz Gökengin

Peter Goldblatt

Beatriz González López-Valcárcel

Michael Goodman

Magnus Gottfredsson

Rebecca Gould

Stefano Govoni

Igor Grabovac

Alice M Grady

Norbert Graf

Michael James Green

Claire Greene

Jostein Grimsmo

Steven K Grinspoon

Martin Grobusch

Jan Groen

Margreth Grotle

Maurizio Gualtieri

Giovanni Guaraldi

Sebastian Guenther

Bruno Guerci

Stéphanie Guey

Alicia Guillien

Aakriti Gupta

Nils Gutacker

Elspeth A Guthrie

Francisco Miguel Gutierrez-Mariscal

Thomas Haak

Marcus Hacker

Allan Hackshaw

Dieter Hahnloser

Helinä H Hakko

Christian Hakulinen

Nigel Hall

Gerhard Hamann

William Hamilton

Adam E Handel

Benjamin Handen

Paul Hanly

Thomas Harder

Michael Harhay

Diane Harper

Patrick Harris

Rodrigo Hasbun

Henrik Hasle

Christian Hassager

Jeannine L A Hautvast

Hua He

Yulei He

Wenche Hegelstad

Janneke Heijne

Jonas Heitmann

Camilo Helito

Joris Hemelaar

Claire Henderson

Peter Hendricks

Linda L Henry

Julia Hentschel

Daniel Herman

Peter Hermann

Ramon Hermida

Alina Herrmann

Mark Hew

Matthew Hickman

Blánaid Hicks

Takahiro Higashi

Stephen Higgins

Luuk Hilbrands

Fumihito Hirai

Tsunahiko Hirano

Rosemary Hiscock

Gerard Hoek

Bruno Hoen

Georg Hoffmann

Michael Hofman

Carla Hollak

Thomas Holland

Lene Holmvang

Sung-Jin Hong

Jan Hontelez

Peter Hoskin

Nehmat Houssami

Louise Howard

Laura Howe

Liang-Hao Hu

Shi Huang

Niamh Humphries

Philippe Huot

Lisa Hurt

Mathilde M Husky

Jared Huston

David Hutton

Mari Hysing

Antonio Iannelli

Irena M Ilic

Patrick Ingiliz

Hamish Innes

Ana-Maria Iosif

Bilal Irfan

Masoud Isanejad

Michael Ison

David Jacobs

Vincent Jaddoe

Artur Jacek Jakimiuk

Annika Janson

Dorte Jarbol

Darko Jekauc

David J Jenkins

Seogsong Jeong

Helena C B Jernström

Niels Jessen

Daya Krishna Jha

Mengmeng Ji

Shan Jiang

Markus Joerger

Christoffer Johansen

Takeshi Johkoh

Kathryn Johnson

Emily E Johnston

Christine Joisten

Claudio Jommi

Daryl Jones

Jefferson Jones

Kelvin P Jordan

Susan Jordan

Bastien Joubert

Elmar Joura

Claus Juhl

Steven Julious

Sandra Juul

Andrew Kaczynski

Angela Kaindl

Hiltraud Kajüter

Dimitri Kalavrouziotis

Andre Kalil

Roope Kallionpää

Mona Kanaan

Rama Kandasamy

Sasikiran Kandula

Eleanor V Kane

Danbee Kang

Harsh A Kanhere

Vojko Kanič

Hannu Kankaanranta

Stephen Kaptoge

Christian Karagiannidis

Nena Karavasiloglou

Stefan Kasper

Gertjan Kaspers

Srinivasa Katikireddi

Niki Katsiki

Verena Andrea Katzke

Peter Katzmarzyk

Howard Kaufman

Padma Kaul

Paul Keall

Pamela Kearns

Victoria Kehl

Mark Kelson

Zoe Kelson

Friederike Kendel

Wayne Kepner

Winfried Kern

Line Kessel

Aadil Khan

Aditya Khanna

Ali Khashan

Myriam Khlat

Yet Khor

Christine Khosropour

Gregor Kiesewetter

Joosup Kim

Min Chul Kim

Ole Kirk

Sverre Erik Kjeldsen

Knut-Inge Klepp

Kevin J Klos

Sebastian Kluesener

Vitalii Klymchuk

Peter R Knapp

Sebastian Kobold

Michael Kölch

Rositsa G Koleva-Kolarova

Yoshito Komatsu

Evangelos Kontopantelis

Karel Kostev

Konstantinos Kostikas

Chelsea Kotch

Oswald Kothgassner

Konstantinos Kotsis

Ilse Kouijzer

V P Kovalchuk

Justyna Kowalska

Robert Krause

Rafał Krenke

Anand Krishnan

Leonard Kritharides

Andreas Kronbichler

David Krug

Kiersten Kugeler

Roland Kuiper

Deepali Kumar

Toshiki Kuno

Hannah Kuper

Lukasz Kuryk

Oliver Kuss

Matthew Y W Kwan

Alessio Lachi

Joseph A Ladapo

Lies Lahousse

Jimmy Che-To Lai

Botond Lakatos

Sham Lal

Pier Lambiase

Andre Lamy

Weizhong Lan

Jaap Lancee

Thorsten Langer

Niklas Langstrom

Natasha A Lannin

Therese Sophie Lapperre

Francesca Larese Filon

Matthew Michael Large

Britta A Larsen

Angioletta Lasagna

Eric Lau

Elise Launay

Jeffrey Lazarus

Carel le Roux

Christine Lebrun Frenay

Fiona Lecky

Alvin Lee

Eun-Young Lee

Wei-Ju Lee

Y C Gary Lee

Rolf Lefering

Jiayao Lei

Marc Leone

Yossi Levi-Belz

Robert D Levitan

Stuart Levitz

Dan Lewer

Nathaniel Lewis

Victoria Lewis

Joel Lexchin

Mengtao Li

Xingnan Li

Evangelos Liberopoulos

Øjvind Lidegaard

Fabian Liechti

Matthias Liechti

Annette Lim

Youn-Hee Lim

Direk Limmathurotsakul

Giuseppe Limongelli

Nancy W Lin

Anna Lindholm-Olinder

Rachel Lindor

Daniel Lindqvist

Suping Ling

Senthil Lingaratnam

Lynda Lisabeth

Chen Liu

Gang Liu

Po-Yu Liu

Zhi-Peng Liu

Marc B I Lobbes

Rakesh Lodha

Marie Löf

Marek Lommatzsch

Sara Lönn

Pietro Luigi Lopalco

Roberto Lorusso

Paul Loubet

Alexandre Loupy

Marianne Lovstad

Madia Lozupone

Brigitte Lueger-Schuster

Hao Luo

Elsebeth Lynge

Georgios Lyratzopoulos

Mark Lythgoe

Wenxue Ma

Colin Francis Macdougall

Ausenda MacHado

Dorothy Machalek

Rocio Macias

Graeme MacLennan

Peter MacPherson

Guy J Maddern

Sten Madsbad

Mads Alexander Just Madsen

Michael Maeng

Nyashadzaishe Mafirakureva

Maria Christine Magnus

Emmanuella Magriplis

Rajiv Mahajan

Alyson L Mahar

Maria Ida Maiorino

Afshin Maleki

Alex Manara

Gilles Manceau

Olivia Manfrini

Kevin Mani

Antonino Maniaci

Gregory Mann

Mohammad Ali Mansournia

Adel Mansur

Sara Manti

Evangeline Mantzioris

Paolo Manzoni

Giulio Marchesini

Raffaele Marfella

Alessandro Marinaccio

Giovanni Marini

Giovanni Mariscalco

Michel Marre

John Marshall

Tom Marshall

Vassilis Martiadis

Tero Martikainen

Cara Martin

Cristina Martín Lorente

Benedict Martina

Erika Martinelli

Dmytro Martsenkovskyi

Florian Marx

Atsushi Masamune

Nick Maskell

Gilbert Massard

Ben Mathews

Rubeta Matin

Koji Matsuo

Kazuhide Matsushima

Alberto Matteelli

Barbara Maughan

Robert Gordon Maunder

Victor Mauri

Didac Mauricio

Konstantinos Mavridis

Marc Maynadié

Daniel McCartney

Linda K McEvoy

Rebecca McKetin

Luke S McLean

Meredith McMorrow

Amy McNeilage

Enkeleint A Mechili

Maria Melchior

Dominik Menges

Fiona Mensah

Nicolas Menzies

Tracy L Merlin

Annemarie Messchendorp

Sven Günther Meuth

Patrick Meybohm

Haakon Meyer

Patrick M Meyer Sauteur

Christine Miaskowski

Cathrine Mihalopoulos

Tuija Mikkola

Antonina Mikocka-Walus

Rafael Mikolajczyk

Portia L Miller

Rachel Miller

Geltrude Mingrone

Alexander Dimitri Miras

Viktar Mitsura

Hidehiro Mizusawa

Valentin Mocanu

Ashley Moffett

Igor Mokrousov

Javier Molina

Ann Merete Møller

Susana Monge

Mastura Monif

Catrin Moore

David Moore

Marco Morabito

Paul Moran

Mario Luca Morieri

Lara Morley

Barbara Mostacci

Paul Mouncey

Bruno Mourvillier

Zarah Moussavi

Alexander Muela

David Murdoch

Gavin J Murphy

Martha Meaney Murray

Nicholas Murray

Cristina Mussini

Paul Myles

Martin Mynarek

Peter Na

Fardin Nabizadeh

Simon Nadel

Gilles Naeije

Mohsen Naghavi

Anatol-Fiete Näher

Atsushi Nakajima

Sandip Nandhra

Valerio Nardone

Eleni Nastouli

Jean–Charles Nault

Fernando Navarro-Mateu

Sergiusz Nawrocki

János Németh

Kirsten Ness

Rinze Neuteboom

Gilles Nevez

Alicia Nevriana

Jeffrey Newcorn

Edmond Ng

Emanuele Nicastri

Federico Nichetti

Stephen Nicholls

Angela M Nickerson

Susanne Nielsen

Suzanne Nielsen

Teemu Niiranen

Elena Nikiphorou

Roy Miodini Nilsen

Sisse Helle Njor

Wanjikũ Njoroge

Peter Noordzij

Merete Nordentoft

Jacopo Nori

John Norrie

Jean Baptiste Nousbaum

Amy Nunn

Marianne Nygaard

Malin Nygren-Bonnier

Sandra N Ofori

Nicholas Ogden

Tetsu Ohnuma

Kevin Kris Warnakula Olesen

Timothée Olivier

Jonathan Olsen

Jason Ong

Marco Onofrj

Daniel Ontaneda

Bregje Dorien D Onwuteaka-Philipsen

Felix Onyije

Corentin Orvain

Fabienne Ory-Magne

Michael Osborn

David Osrin

Marlies Ostermann

Ignacio Oulego

Nina Cecilie Øverby

Volkan Özenci

Inês Paciência

Andrzej Pająk

Amir Haji Agha Pakpour

Diego Palumbo

Margarita Panayiotou

Damiano Paolicelli

Helle Pappot

Yann R Parc

Milosz Parczewski

Manish Pareek

Lucilla Parnetti

Guido Parodi

Pier Camillo Parodi

Toshima Parris

Stephanie Partridge

Patrizio Pasqualetti

Francesco Passiglia

Jakob Passweg

Sanjay Patole

François Pattou

Iuliia Pavlova

Timothy Pawlik

Lillian Pazvakawambwa

Rupert Pearse

Markus Peck-Radosavljevic

Alma Pedersen

Marijke Peetermans

Tuula Pelkonen

Ville Peltola

Sirkku Peltonen

José L Peñalvo

Sarah Pendlebury

Prince Peprah

Mónica Pérez-Ríos

Zane B Perkins

Carlo Federico Perno

Maiju Pesonen

Eskild Petersen

Nicola Petrosillo

Guenka Ivanova Petrova

Isabelle Peytremann-Bridevaux

Nicole R Phillips

Fredrik Piehl

Alessandra Pierangeli

Sante Donato Pierdomenico

Jean-Yves Pierga

Angelo Pietrobelli

Irina Pinchuk

Marta Pingarilho

Matthias Pinter

Antonio Piralla

Claudia Pisanu

Oleguer Plana-Ripoll

Davor Plavec

Saskia Pluijm

Daria N Podlekareva

Paul Poirier

Maurizio Pompili

Barry Popkin

Laurent Portel

Louis Potier

Neil R Poulter

Carlo Pozzilli

Barbara Prainsack

Robin Prescott

Ulrich Wolfgang Preuss

Susan Prockop

Giuseppe Procopio

Alessandra Protti

Wendi Qian

Terence Quinn

Carlos R Quiñonez

Jennifer Quint

Carlos Rabade

Angelo Roberto Raccagni

Peter Rafaj

Chantal Raherison-Semjen

Mohamed Rahouma

Giovanni Raimondo

Mika Ramet

Sriram Ramgopal

Jose-Manuel Ramos-Rincon

Nicole Rankin

Gerhard N Ransmayr

Otavio Ranzani

Daiva Rastenyté

Gerry Rayman

Neda Razaz

Oliver Razum

Paola Rebora

Rita Redberg

Jaap C Reijneveld

Kyndaron Reinier

Sebastian Johannes Reinstadler

Steffen Rex

Julia Rey Brandariz

Ciara Reynolds

Abanoub Riad

Matteo Riccò

Lucie Richard

Elvira Richter

Florian Rieder

Damian Rieke

Alan S Rigby

Cristina Rius

Alessandro Rizzo

Andrea Roberts

Chris Robertson

Ada Margarida Correia Nunes Rocha

Ana Paula Rodrigues

Kris Rogers

Eliane Rohner

Sabine Rohrmann

David Rojas-Rueda

Martina Rojnic Kuzman

Giulio Francesco Romiti

Gholamreza Roshandel

Hana Ross

Wolfgang A Rottbauer

Jessica Roydhouse

Julian Rubel

Alessandra Rufa

Massimo Rugge

Annalisa Ruggeri

Jan Rupp

Ruediger Rupp

Mark Russell

Defne Saatci

Michela Sabbatucci

Séverine Sabia

Francesco Sacca

Manish Sadarangani

Mohammad Reza Saeb

Marc Saez

Apostolos Safouris

Marina Sagud

Philippe Saiag

Atsuhito Saiki

David P Salmon

Lucy Salmon

Amber Salter

Jerzy Samochowiec

Kristian Samuelsson

Pablo José Sánchez

Jane Sandall

Johanna Sandgren

Charbel Sandroussi

Raul Santos

Grant E Sara

Alain Saraux

Koji Sasaki

Daniel Satge

Gregory R Savage

Susan Sawyer

Andrea Schaffer

Maarten Schim van der Loeff

Sandra Schlegl

Ekkehard Schleußner

Moritz Schmelzle

Michael Rahbek Schmidt

Andreas Schnitzbauer

Robert Schoevers

Tobias Schupp

Matthias Schwab

Bjoern Schwander

Robert Schwartz

Michaël Schwarzinger

Daniel Schwarzkopf

Giorgio Scivoletto

Martin Sebastian

Farah Seedat

Jean M Seely

Petar Seferović

Leonie Segal

Hubert Seggewiss

Samuel Seidu

Abhijit Sen

Stefano Sensi

Gianluca Serafini

Walter Sermeus

Laurent Servais

Marianne Severinsen

Wissam Shalish

Huifang Shang

Michael Shapiro

Michelle W Sharp

Robert Sheldon

Guofeng Shen

Zaki Sherif

Jodi Sherman

Lizheng Shi

Neal Shore

Takehito Shukuya

Pierre Sicard

Noriane Sievi

Katarzyna Sikorska

Camillo Silibello

Gregory Simon

Colin Simpson

Shireen Sindi

Daisy Singla

Nirmish Singla

Susan B Sisson

Magnus Sjögren

Halyna Skipalska

Helen Skouteris

Jared Smith

Rebecca Smith-Bindman

Helen Snooks

Francesco Sofi

Olusegun Soge

Roy L Soiza

Maria José Soler Romeo

Renata Solimini

Jin Woo Song

Jing Song

Romain Sonneville

Silvia Sookoian

Sveinung Sørbye

Vicente Soriano

Antoni Soriano-Arandes

Veronica Southerland

Teresa Spadea

Paolo Spagnolo

Christoph Spinner

Robert Spinner

Tanja Sprave

Raj Srirajaskanthan

Paddy Ssentongo

Adam Staffaroni

Stephen Stansfeld

Tom Stargardt

Sarah Steeg

Norbert Stefan

Peter Stehle

Giulia Stella

Erik Stenberg

Olof Stephansson

Matt Stevenson

Robert Stewart

Gregg Stone

Stefan Störk

Jacob Strahilevitz

Tessa Strain

Kathrin Strasser-Weippl

Ulf Strömberg

Jan Styczyński

Tung-Hung Su

Ralf Sudbrak

Yuelian Sun

Rajeshwari Sundaram

Antonio Suppa

Jannet Svensson

Umang Swami

Peter Swoboda

Tamas Szakmany

Tibor Szarvas

Yen Foung Tai

Derrick Tam

Kelvin Bryan Tan

Hajime Tanaka

Shinji Tanaka

Su'an Tang

Vin Tangpricha

Pieter Tanis

Philip Tarr

Christoph Johannes Tausch

Vicky Taxiarchi

Katja Taxis

Roy Taylor

Tobias Tenenbaum

Joan M Teno

Claudio Terranova

Tomas Tesar

Anaïs Teyton

Holger Thiele

Stephanie Thomas

Reimar Thomsen

Anne Amalie Elgaard Thorup

Olena Tigova

Shane Tillakeratne

Lars Timmermann

Rania Tohme

Eleonora Topino

Michelle Torok

José David Torres-Peña

Richard Tothill

Dario Trapani

Santi Trimarchi

Palak Trivedi

Nico Trocmé

Joan Carles Trullàs

Konstantinos Tsilidis

Georgios Tsivgoulis

Stephanie Tubert-jeannin

Patricia Tucker

Pauline Turnbull

Katy Turner

David K Turok

Christopher P Twine

Tanja Adele Tydén

Alp Ucok

Kennichiro Uemura

Marleen Van Baak

Thomas P Van Boeckel

Niels van de Donk

David van de Vijver

Chris van den Akker

Roderick C N Van Den Bergh

Bert Jan H van den Born

Michiel van der Flier

Lorenz Van der Linden

Jelle van der List

Jan van der Meulen

Marc van der valk

Karin van Dijk

H Rogier van Doorn

Frederieke H van Duijnhoven

Thilo van Eimeren

Mieke Van Hemelrijck

Annemieke van Straten

Gianluca Vanni

Amanda M Varnava

Marc Vasse

Monica Vavilala

Latha Velayudhan

Balasubramanian Venkatesh

Robert Verdonk

Guido Veronese

Monique Verschuren

Josef Veselka

Davide Liborio Vetrano

Marie Vidailhet

Samuel Videholm

Anita Villani

Jesus Villar

Tim O Vilz

Marianna Virtanen

Rozemarijn Vliegenthart

Dominique Vodovar

Kerstin Noëlle Vokinger

Massimo Volpe

Clemens von Birgelen

Uwe von Fritschen

Peter von Philipsborn

Alex Vorsters

Athanasios Voulgaris

Marija Vukoja

Renske I Wadman

Steven Waguespack

Marlies Wakkee

Fredrik Walby

Maciej Walędziak

Josephine Walker

David Walsh

Emily S Wan

Johannes Wancata

Per Wandell

Fenglei Wang

Jaw-Yuan Wang

Juan Wang

Joanna Wardlaw

Richard Watt

Mayyada Wazaify

Gwilym Webb

Michael Weichenthal

Brian Weir

Paul Weiss

Martin Wermke

Jasmin Wertz

Keith West

Marcus Westerberg

Björn Wettermark

Jack F M Wetzels

Harvey White

Gunnar Wichmann

Kolitha Wickramage

Christian Wiedermann

Katarzyna Wiktorska

Sarah Wild

Joanne Wildenbeest

Malgorzata Wilinska

Katalin Wilkinson

Robert Wilkinson

Samantha Willan

John Williams

Thomas Williams

Anders Wimo

Petr Winkler

Anna Winkvist

Lilian Witthauer

Arnold Wong

Kitty H F Wong

Ngai Sze Wong

Catherine B Woods

Roelie Wösten-van Asperen

Albert Wu

Jason Wu

Jianhua Wu

Rachel Wuerstlein

Ningshao Xia

Meng Xiao

Shao-Hua Xie

Leshan Xiu

Xiaolin Xu

Zhiwei Xu

Sıddika Songül Yalçın

Ming Yang

Ting Yang

Sheraz Yaqub

Lei Ye

Joseph Yeboah

Peng Yin

Guishuang Ying

Janelle A Yorke

Aisha Yousafzai

Bo Yu

Jin-Tai Yu

Ke-da Yu

Oriana Hoi Yun Yu

Vesna Zadnik

Galia Zamaratskaia

Antonis Zampelas

Dorota Zarębska-Michaluk

Hajo Zeeb

Sylke Zeissig

Alexei Zelenev

Florian Zepf

Líbia Zé-Zé

Xiaoxi Zhang

Fengmin Zhao

Guoguang Zhao

Shuguo Zheng

Tao Zhou

Hui Zhu

Wengen Zhu

Thomas Zilli

Martha Zimmermann

Ariana Znaor

Marco Zuin

Maëva Zysman

